# Chronic stress leading gut microbiota dysbiosis and neuroinflammation promoted depressive disorder

**DOI:** 10.1038/s41398-026-04421-8

**Published:** 2026-09-01

**Authors:** Xinhua Shen, Xiaofeng Jiang

**Affiliations:** Shaoxing Seventh People’s Hospital(Affiliated Meatal Health Center, Medical College of Shaoxing University), Shaoxing, China

**Keywords:** Depression, Molecular neuroscience

## Abstract

Chronic stress was known to contribute to gut microbiota dysbiosis and central neuroinflammation, yet the underlying mechanisms remain poorly understood. In this study, forty-eight male C57BL/6 mice were randomly assigned to three groups: control, chronic unpredictable mild stress (CUMS), and CUMS with probiotic intervention. Mice in the CUMS group were exposed to four weeks of unpredictable stressors, while those in the probiotic group received oral administration of Bifidobacterium triple viable capsules during the stress period. Behavioral assessments revealed pronounced depression-like behaviors in CUMS mice, which were significantly ameliorated by probiotic treatment. 16S rRNA sequencing demonstrated that CUMS reduced gut microbial α-diversity, altered β-diversity, elevated the Firmicutes/Bacteroidetes ratio, and decreased beneficial genera such as Bifidobacterium and Lactobacillus, while increasing the abundance of Escherichia. Probiotic supplementation effectively restored these microbial alterations to near-control levels. Intestinal barrier function was compromised in CUMS mice, as indicated by reduced proteins ZO-1 and Occludin, which partially reversed by probiotic treatment. In the hippocampus and prefrontal cortex, CUMS increased the levels of pro-inflammatory cytokines (IL-1β, TNF-α, and IL-6), and upregulated microglial activation markers (Iba-1, iNOS, and COX-2) in the hippocampus. Probiotic intervention markedly suppressed these neuroinflammatory responses. Moreover, hippocampal CA1 neurons in CUMS mice exhibited structural disorganization, neuronal loss, and nuclear pyknosis, all of which were alleviated by probiotic administration. Collectively, these findings suggest that chronic stress disrupts the gut microbiota–gut–brain axis via microbial dysbiosis and intestinal barrier impairment, thereby promoting neuroinflammation and behavioral abnormalities.

## Introduction

Chronic stress is a persistent physiological or psychological state that activates the hypothalamic-pituitary-adrenal axis and sympathetic nervous system, leading to multi-system dysfunction and a substantially increased risk of depressive disorders with poorer prognosis [1–3]. As the monoamine hypothesis cannot fully explain the specific molecular mechanisms of stress-induced psychiatric disorders, research has shifted toward the roles of gut microbiota and inflammatory responses.

The gut microbiota, the body’s largest microbial community, is integral to nutrient metabolism and immune regulation, communicating with the central nervous system via the gut-brain axis through neural, humoral, and immune pathways [4, 5]. For example, depressed patients exhibit gut microbiota dysbiosis, including an elevated Firmicutes/Bacteroidetes ratio and reduced abundance of beneficial genera such as Bifidobacterium and Lactobacillus, suggesting microbial involvement in affective disorders [6].

Chronic stress also activates microglia, increasing pro-inflammatory cytokines like IL-1β, TNF-α, and IL-6, which can impair neurons by disrupting neurotransmitter synthesis, synaptic plasticity, and promoting apoptosis, thereby disturbing emotional regulation [7, 8]. Clinically, elevated pro-inflammatory cytokines in depression correlate with symptom severity [9].

Stress-induced dysbiosis can compromise intestinal barrier integrity, permitting bacterial translocation and systemic dissemination of pathogen-associated molecular patterns such as lipopolysaccharide, which promotes low-grade inflammation that may cross the blood-brain barrier to affect the central nervous system [10]. Neuroinflammation may in turn alter gut microbiota via the gut-brain axis, establishing a vicious cycle of microbiota dysbiosis and neuroinflammation [11]. However, the precise molecular interactions between these systems under chronic stress remain unclear and lack direct experimental validation.

Using a chronic unpredictable mild stress mouse model with probiotic intervention, this study systematically examines how chronic stress alters gut microbiota and intestinal barrier function, induces neuroinflammation, and mediates crosstalk between dysbiosis and neuroinflammation, while evaluating how microbiota modulation improves inflammatory and behavioral outcomes through behavioral, microbiological, molecular, and histopathological analyses. This work aims to clarify gut-brain axis mechanisms in stress-related psychiatric disorders, offering evidence for microbiota-targeted interventions.

## Methods

### Experimental animals, reagents and instruments

#### Experimental animals

Forty-eight SPF grade C57BL/6 male mice, aged 6-8 weeks and weighing 20–22 g, were purchased from Chongqing Model Animal Resource Center.The sample size was estimated based on preliminary pilot data and standard practices in preclinical research to minimize animal usage while ensuring statistical validity. All mice were housed in an SPF-grade barrier environment at the Animal Facility of Chongqing Medical University. Housing conditions included a temperature of 22 ± 2 °C, relative humidity of 55 ± 5%, and a 12-hour light/dark cycle (light period: 07:00-19:00). The mice had free access to sterile water and standard rodent chow. After one week of acclimatization, experiments commenced. All procedures were in accordance with the National Institutes of Health Guidelines for Animal Research (Guide for the Care and Use of Laboratory Animals) and were approved by the Animal Ethics Committee of Chongqing Medical University.

#### Reagents

Bifidobacterium Triple Live Capsules (containing Bifidobacterium, Lactobacillus acidophilus, and Enterococcus faecalis; live bacterial count ≥ 1.0 × 10⁹ CFU per capsule; Shanghai Xinyi Pharmaceutical Factory); Mouse IL-1β, TNF-α, and IL-6 ELISA kits (R&D Systems, USA); Primary antibodies for ZO-1, Occludin, Iba-1, iNOS, COX-2, and GAPDH (Cell Signaling Technology, USA); HRP-labeled secondary antibodies (Beijing Zhongsan Jinqiao Biotechnology Co., Ltd.); Immunohistochemistry kits (Beijing Vector Laboratories); 16S rRNA gene sequencing kit (Shanghai Meiji Biomedical Technology Co., Ltd.); Total protein extraction kit and BCA protein quantification kit (Nanjing Kaiji Biotechnology Co., Ltd.). Other routine chemical reagents were all of analytical grade and purchased from Sinopharm Chemical Reagents Co., Ltd.

#### Instruments

Animal Behavior Analysis System (Shanghai Xinruan Information Technology Co., Ltd., XR-XZ301); High-throughput Sequencer (Illumina, USA, MiSeq); Microplate Reader (Bio-Rad, USA, Model 680); Western blot Electrophoresis and Transfer System (Bio-Rad, USA, Mini-PROTEAN Tetra); Fluorescence Microscope (Olympus, Japan, BX53); Paraffin Sectioner (Leica, Germany, RM2245); Low-Temperature High-Speed Centrifuge (Eppendorf, Germany, 5810R); Ultra-low Temperature Freezer (Thermo Fisher, USA, −80 °C).

### Experimental grouping and treatment

#### Group design

**Fig. 1:**
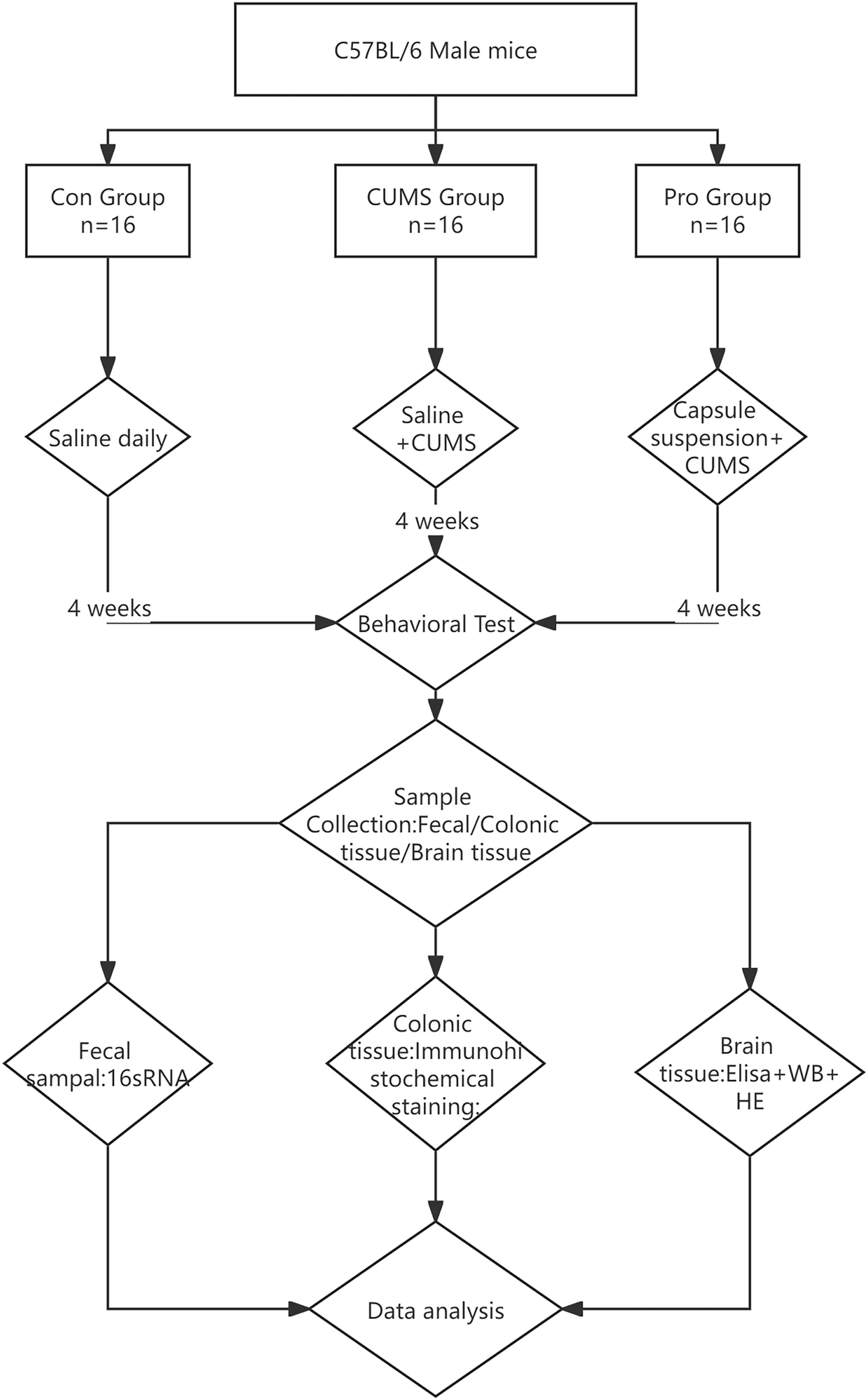
Protocol. This is the flowchart of the entire study, which includes steps such as model construction, intervention, sampling, and analysis.

Forty-eight mice were randomly divided into three groups using a random number table method, with 16 mice in each group: 1. Control Group (Con Group): Routinely reared without any stress treatment, receiving intragastric administration of equivalent volumes of saline daily; Chronic Unpredictable Mild Stress group (CUMS group): Stress models were established using the CUMS method, with daily intragastric administration of an equivalent volume of physiological saline. Probiotic Intervention group (Probiotics Group): During CUMS stress exposure, mice received daily intragastric administration of Bifidobacterium Triple Viable Capsule suspension (Fig. 1).

#### Establishment of CUMS model

Adapted from the method by Zhang et al. [12] with modifications, the stress cycle lasted 4 weeks. One to two stressors were randomly selected daily to prevent habituation: 1. Food deprivation for 24 hours; 2. Water deprivation for 12 hours; 3. Cage tilting (45°) for 24 hours; 4. Damp bedding (bedding soaked in sterile water) for 24 hours; 5. Light/dark cycle reversal (12-hour light/12-hour dark inversion); 6. Social isolation (single-cage housing) for 24 hours; 7. Mild electric shock (0.5 mA current, lasting 1 s, at 10 s intervals, 30 times total). Mice in the Control Group were housed together with the stress group to avoid olfactory interference and did not undergo stress treatment.

#### Probiotic intervention protocol

Bifidobacterium Triple Viable Capsules were prepared into a suspension using sterile saline at a concentration of 1×10⁹ CFU/mL. Starting from day 1 of stress exposure, mice in the Probiotics Group received daily intragastric administration at 9:00 AM at a dosage of 10 mL/kg body weight for 4 consecutive weeks. The Control Group and CUMS group received equal volumes of sterile saline via intragastric administration, following the same procedure as the Probiotics Group.

### Behavioral test

All behavioral tests were conducted within 24 hours after stress exposure. The test sequence was as follows: Open Field Test → Elevated Plus Maze Test → Forced Swim Test. Each test was separated by a 24-hour interval. Testing environment was maintained in quiet conditions with temperature controlled at 22 ± 2 °C. All experiments within the same batch were performed by the same experimenter.Blinding was not performed during the behavioral assessments and data analysis.

### Sample collection and processing

Within 24 hours after behavioral testing, anesthetize the mice by intraperitoneal injection of 1% pentobarbital sodium (50 mg/kg): Fecal samples: Collect fresh feces from mice under sterile conditions, place them in enzyme-free centrifuge tubes, and immediately store at -80 °C for 16S rRNA gene sequencing; Colonic tissue: Dissect and isolate mid-colon tissue (approximately 0.5 cm). Fix one portion in 4% paraformaldehyde solution for immunohistochemical detection. Preserve another portion in enzyme-free centrifuge tubes at −80 °C for subsequent use; Brain tissue: Rapidly isolate the brain, collect hippocampal and prefrontal cortex tissues, place them separately in enzyme-free centrifuge tubes, and store at −80 °C for ELISA and Western blot analysis. Intact brain tissues were fixed in 4% paraformaldehyde solution for HE staining.

### Testing methods

#### Gut microbiota 16S rRNA gene sequencing analysis

The analysis was commissioned to Shanghai Majorbio Bio-Pharm Technology Co., Ltd. 1. Fecal sample microbial genomic DNA extraction: Total DNA was extracted using the CTAB method, with DNA purity and integrity verified by 1% agarose gel electrophoresis. 2. 16S rRNA gene amplification: Using DNA as template, the V3-V4 region of bacterial 16S rRNA gene was amplified with primer sequences: 338F (5’-ACTCCTACGGGAGGCAGCAG-3’), 806R (5’-GGACTACHVGGGTWTCTAAT-3’). 3. Library Construction and Sequencing: Purified PCR products were used to construct Illumina libraries, followed by paired-end sequencing on the MiSeq platform. Bioinformatics Analysis: QIIME2 software was employed for sequence quality control, assembly, and clustering to obtain Operational Taxonomic Units (OTUs). Analyses included α-diversity (Shannon index, Simpson index), β-diversity (PCoA analysis), as well as phylum- and genus-level microbial composition and relative abundance.

#### Immunohistochemical detection of tight junction proteins in colonic tissue

Preparation of Paraffin-Embedded Sections: Colonic tissues were fixed in 4% paraformaldehyde for 24 h, followed by gradient dehydration, transparency processing, paraffin embedding, and continuous sectioning at 5μm thickness. Immunohistochemical staining: After deparaffinization and rehydration, endogenous peroxidase was blocked by incubating with 3% H₂O₂ at room temperature for 10 min. Antigen retrieval was performed using citrate buffer (pH 6.0) under high-temperature conditions. Sections were blocked with 5% BSA for 30 min, followed by overnight incubation at 4 °C with primary antibodies (ZO-1 1:200, Occludin 1:200). After three PBS washes, HRP-labeled secondary antibodies were added and incubated at room temperature for 30 min. DAB was used for chromogenic development, hematoxylin for counterstaining, and sections were dehydrated, cleared, and mounted with mounting medium; Result analysis: Under fluorescence microscopy, five random sections per group were selected with five fields of view (×400) per section. The Mean optical density value (MOD) was calculated using ImageJ software to reflect protein expression levels.

#### Inflammatory cytokine ELISA assay

Sample processing: Frozen hippocampal and prefrontal cortex tissues were accurately weighed and homogenized in ice-cold PBS buffer (containing Protease Inhibitor) at a strict weight-to-volume ratio of 1:10 (w/v, e.g., 10 mg tissue per 100 μL buffer) under ice-bath conditions. To ensure the comparability of cytokine levels across different groups, all homogenates were prepared using this identical ratio. Consequently, the cytokine concentrations measured in the supernatants (pg/mL) are directly proportional to the absolute content per unit mass of the tissue (pg/mg tissue). Total protein normalization was not performed. The homogenates were centrifuged at 4 °C at 12,000 rpm for 15 min, and supernatants were collected; 2. Detection procedure: Following the ELISA kit manufacturer’s instructions strictly, standard wells, blank wells, and sample wells were set up. Standards and samples were added followed by 90-min incubation at 37 °C. After washing, enzyme-labeled antibody was added and incubated at 37 °C for 60 min. After another wash, chromogenic substrate was added and incubated at 37 °C in the dark for 15 min. The reaction was terminated by adding stop solution; 3. Results analysis: Absorbance (OD value) at 450 nm wavelength was measured using a microplate reader. Concentrations of IL-1β, TNF-α, and IL-6 in samples were calculated based on the standard curve.

#### Western blot detection of hippocampal tissue

Total protein extraction: Frozen hippocampal tissue was homogenized in RIPA lysis buffer (containing protease inhibitors and phosphatase inhibitors) at a 1:10 ratio via ice-bath homogenization. After centrifugation at 12,000 rpm for 15 min at 4 °C, the supernatant was collected; 2. Protein quantification: Protein concentration was determined using the BCA method. Protein loading amounts were adjusted according to quantification results; 3. SDS-PAGE Electrophoresis and Membrane Transfer: 50 μg protein samples were separated by 10% SDS-PAGE electrophoresis. After electrophoresis, proteins were transferred onto PVDF membranes and blocked with 5% skim milk at room temperature for 2 h. Antibody Incubation: Membranes were incubated with primary antibodies (Iba-1 1:1000, iNOS 1:1000, COX-2 1:1000, GAPDH 1:5000) at 4 °C overnight. After three washes with TBST, HRP-labeled secondary antibody (1:5000) was added and incubated at room temperature for 1 h. Detection and Quantification: Protein bands were visualized using an ECL chemiluminescence kit and imaged with an ImageQuant LAS 4000 system. The grayscale ratio of target proteins to internal reference protein (GAPDH) was analyzed using ImageJ software, reflecting the relative expression levels of target proteins.

#### HE staining of hippocampal tissue

Preparation of Paraffin-Embedded Sections: Consistent with the immunohistochemical section preparation method as described; 2. HE Staining: Sections were deparaffinized to water, stained with hematoxylin for 5 min, differentiated in hydrochloric acid-alcohol for 30 s, stained with eosin for 2 min, followed by gradient dehydration, clearing, and mounting with mounting medium; 3. Result Observation: Neuronal morphology in the Hippocampal CA1 region was observed under an optical microscope. Five sections were randomly selected from each group, and three fields of view (×400 magnification) were chosen per section. The number of normal neurons (with intact nuclei, regular morphology, and homogeneous cytoplasm) was counted, and the average number of normal neurons per field of view was calculated.

### Statistical analysis

All experimental data were analyzed and graphed using SPSS 26.0 statistical software and GraphPad Prism 8.0. Quantitative data were expressed as mean ± standard deviation (x̄ ± s). One-way analysis of variance (ANOVA) was employed for multi-group comparisons, with the LSD post hoc test used for pairwise comparisons between groups. β-diversity analysis of gut microbiota was performed using PERMANOVA testing. Categorical data were presented as number of cases (percentage) and analyzed by chi-square test. Statistical significance was defined as P < 0.05. All data meet the normality test requirements.

## Results

### Effects of chronic stress on behavioral outcomes in mice

#### Open field test results

**Fig. 2:**
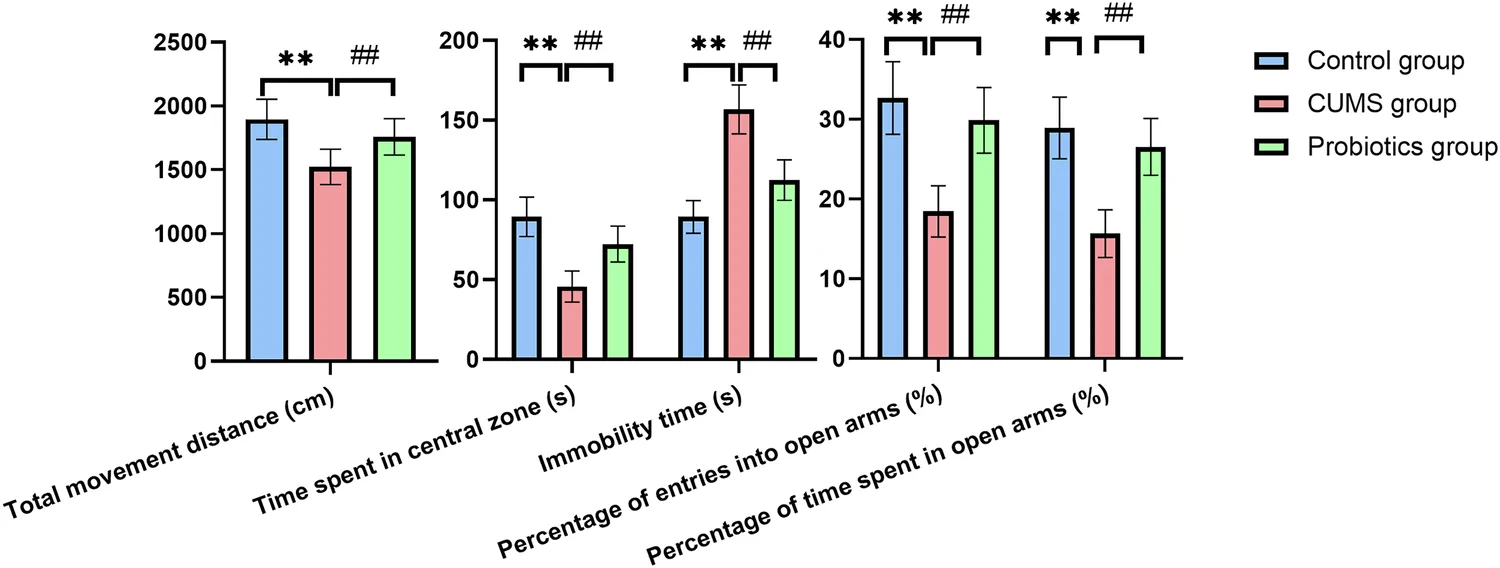
Behavior test results.

Compared with the Control Group (Con), mice in the CUMS group exhibited a significantly decreased total movement distance (P < 0.05) and a markedly shortened time spent in the central zone (P < 0.01). Mice in the Probiotics Group (Pro) showed significantly higher total movement distance and central zone duration than the CUMS group (P < 0.05), but remained lower than the Con group (P < 0.05). These results indicate that CUMS stress leads to reduced spontaneous locomotor activity and exploratory behavior in mice, with probiotic intervention demonstrating partial amelioration of these effects (Table 1, Fig. 2).

**Table 1:** Comparison of behavior test across three groups (mean ± SD, n = 16).

	Control group	CUMS group	Probiotics group
Total movement distance (cm)	1896.32 ± 156.78	1523.47 ± 138.62*	1758.93 ± 142.56#
Time spent in central zone (s)	89.45 ± 12.36	45.67 ± 9.82**	72.34 ± 11.25##
Percentage of entries into open arms (%)	32.67 ± 4.56	18.45 ± 3.21**	29.87 ± 4.12##
Percentage of time spent in open arms (%)	28.93 ± 3.87	15.67 ± 2.98**	26.54 ± 3.56##
Immobility time (s)	89.34 ± 10.25	156.78 ± 15.36**	112.45 ± 12.67##

** Compared with the Control Group, P < 0.01; ##:Compared with the CUMS group, P < 0.01.

#### Results of elevated plus maze test

Mice in the CUMS group exhibited significantly lower percentages of both entries into open arms and time spent in open arms compared to the Control Group (P < 0.01); Both aforementioned indices in the Probiotics Group were significantly higher than those in the CUMS group (P < 0.01), showing no statistically significant differences compared with the Control Group (P > 0.05). The results indicated that CUMS stress could induce anxiety-like behaviors in mice, and probiotic intervention could effectively ameliorate this anxiety state (Table 1, Fig. 2).

#### Results of the forced swim test

The immobility time of mice in the CUMS group during the Forced Swim Test was significantly longer than that in the Control Group (P < 0.01). The immobility time of mice in the Probiotics Group was significantly shorter compared to the CUMS group (P < 0.01), yet remained longer than that in the Control Group (P < 0.05). The results suggest that CUMS stress can induce depression-like behaviors in mice, and probiotic intervention could partially reverse this effect (Table 1, Fig. 2).

### Effects of chronic stress on gut microbiota composition in mice

#### Analysis of gut microbiota α-diversity

**Fig. 3:**
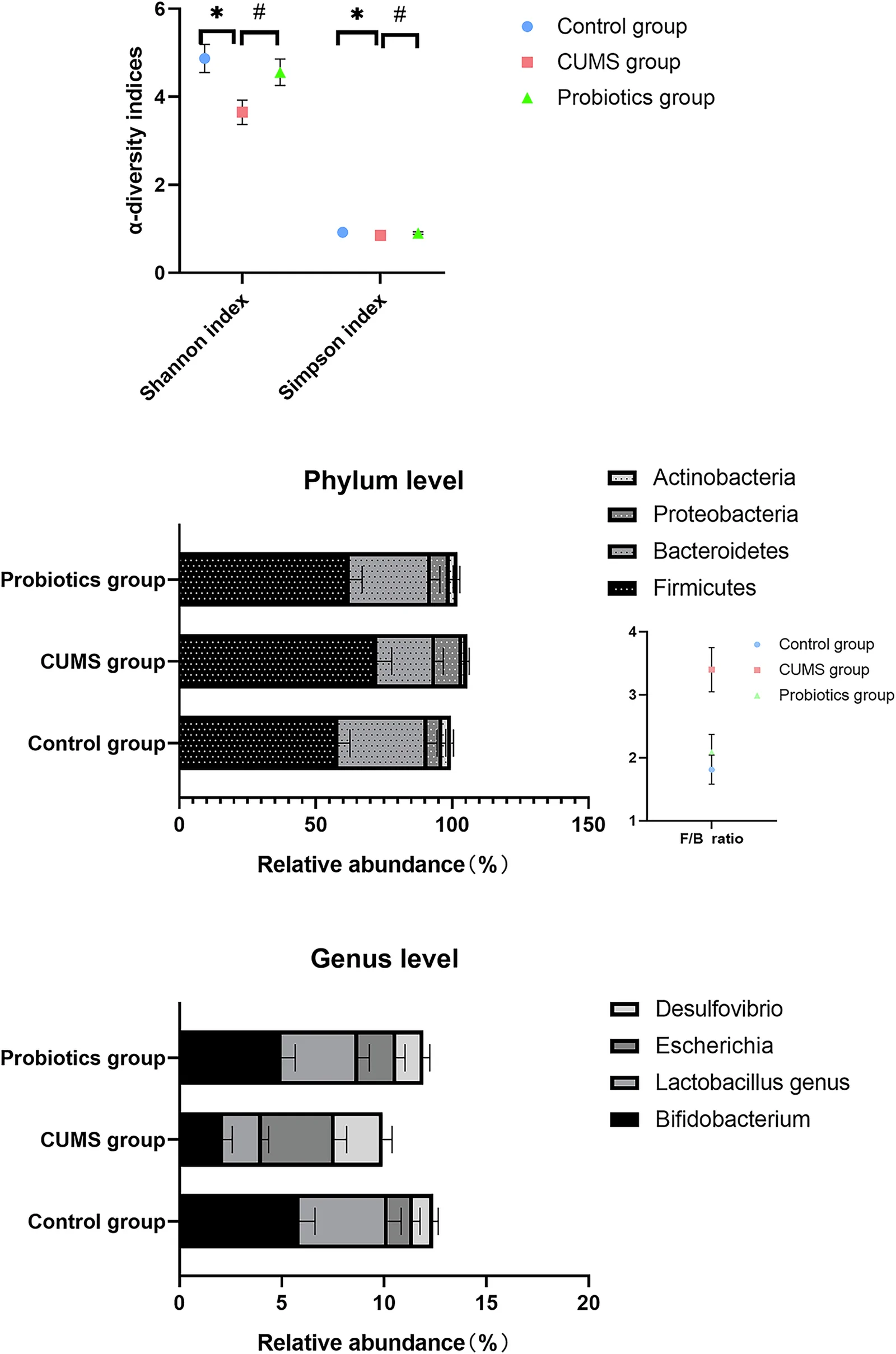
Gut Microbiota composition and relative abundance of three groups.

The Shannon index and Simpson index are important indicators reflecting microbial richness and evenness. Compared with the Control Group, mice in the CUMS group showed significantly decreased Shannon index (P < 0.05) and significantly increased Simpson index (P < 0.05) in gut microbiota; The Probiotics Group exhibited a significantly higher Shannon index compared to the CUMS group (P < 0.05) and a significantly lower Simpson index (P < 0.05), showing no statistically significant differences with the Control Group (P > 0.05). Results indicate that CUMS stress can reduce gut microbiota diversity in mice, while probiotic intervention can restore microbiota diversity (Table 2, Fig. 3).

**Table 2:** Gut Microbiota composition and relative abundance of three groups (mean ± SD, n = 16).

	Control group	CUMS group	Probiotics group
α-diversity indices			
Shannon index	4.87 ± 0.32	3.65 ± 0.28*	4.56 ± 0.30#
Simpson index	0.92 ± 0.03	0.85 ± 0.04*	0.90 ± 0.03#
Relative abundance at phylum level			
Firmicutes (%)	58.34 ± 4.21	72.56 ± 5.34**	62.45 ± 4.56#
Bacteroidetes (%)	32.67 ± 3.56	21.34 ± 2.89**	29.87 ± 3.21#
Proteobacteria (%)	5.43 ± 1.25	9.87 ± 1.56*	6.78 ± 1.34#
Actinobacteria (%)	3.21 ± 0.87	1.89 ± 0.65*	2.98 ± 0.76#
F/B ratio	1.81 ± 0.23	3.40 ± 0.35**	2.09 ± 0.28#
Relative abundance at genus level			
Bifidobacterium	5.87 ± 0.76	2.15 ± 0.43**	4.98 ± 0.68#
Lactobacillus genus	4.32 ± 0.65	1.89 ± 0.32**	3.76 ± 0.54#
Escherichia	1.23 ± 0.34	3.56 ± 0.57*	1.87 ± 0.42#
Desulfovibrio	0.98 ± 0.25	2.34 ± 0.46*	1.32 ± 0.31#

*P < 0.05 vs Control Group, **P < 0.01; #:P < 0.05 vs CUMS group.

#### β-diversity analysis of gut microbiota

**Fig. 4:**
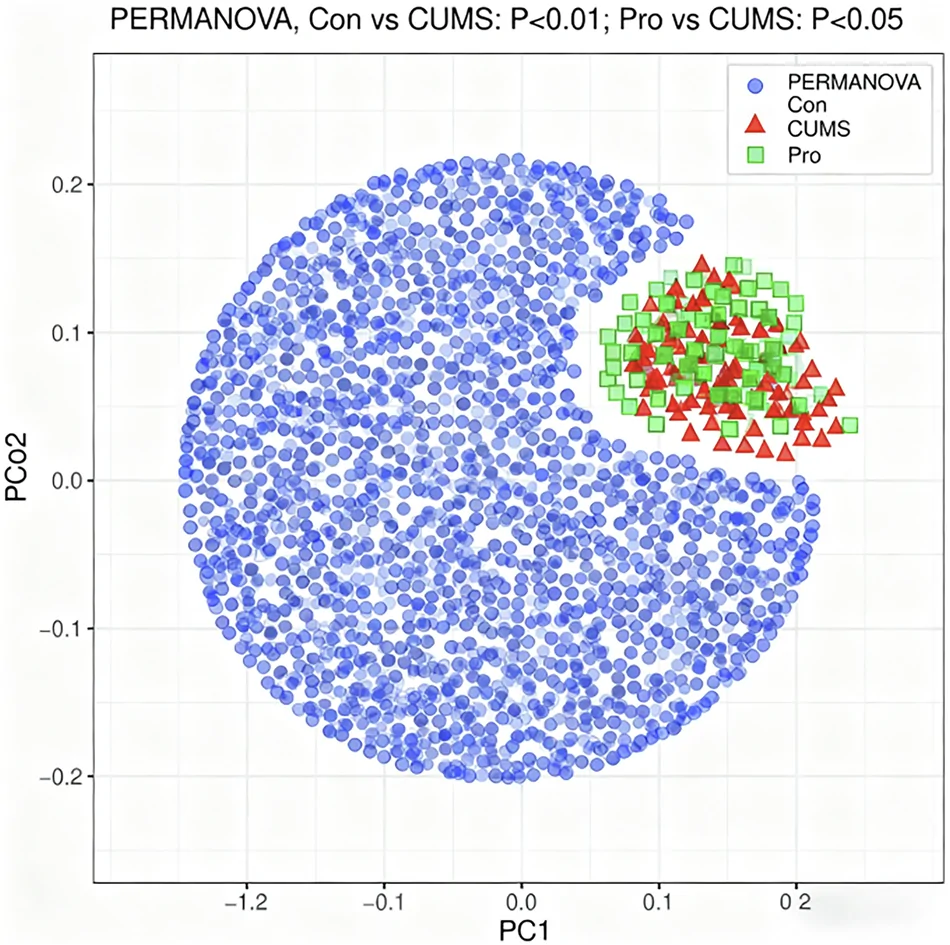
β-diversity analysis of gut microbiota in mice. Principal Coordinate Analysis (PCoA) plot based on the Bray-Curtis distance matrix illustrating the structural differences in microbial communities.Distinct clustering separation was observed between the Control and CUMS groups (PERMANOVA, P < 0.01 P < 0.01). The Probiotics group showed significant separation from the CUMS group (PERMANOVA, P < 0.05 P < 0.05).

PCoA analysis based on the Bray-Curtis distance matrix revealed distinct clustering separation between the gut microbiota samples of the Control Group and CUMS group mice on the PCoA plot (PERMANOVA, P < 0.01), indicating significant differences in microbial composition between the two groups; Samples from the Probiotics Group clustered closer to the Control Group and showed significant separation from the CUMS group (PERMANOVA, P < 0.05), suggesting that probiotic intervention can alter CUMS-induced changes in microbial composition (Fig. 4).

#### Changes in gut microbiota composition at the phylum level

At the phylum level, the gut microbiota of mice was predominantly composed of Firmicutes, Bacteroidetes, Proteobacteria, and Actinobacteria, with Firmicutes and Bacteroidetes being the most predominant (collectively >80%). Compared with the Control Group, the CUMS group showed a significantly increased abundance of Firmicutes (P < 0.01), a significantly decreased abundance of Bacteroidetes (P < 0.01), and a significantly elevated F/B ratio (P < 0.01). Compared with the CUMS group, the Probiotics Group exhibited a significantly reduced abundance of Firmicutes (P < 0.05), a significantly increased abundance of Bacteroidetes (P < 0.05), and a significantly decreased F/B ratio (P < 0.05), with no statistically significant differences compared to the Control Group (P > 0.05) (Table 2, Fig. 3).

#### Changes in gut microbiota composition at the genus level

At the genus level, compared with the Control Group, the CUMS group exhibited significantly reduced abundance of Bifidobacterium and Lactobacillus genus (P < 0.01), while the abundance of Escherichia and Desulfovibrio showed significant increases (P < 0.05). The Probiotics Group showed significantly higher abundances of Bifidobacterium and Lactobacillus compared to the CUMS group (P < 0.05), while exhibiting significantly lower abundances of Escherichia and Desulfovibrio (P < 0.05). No statistically significant differences were observed between the Probiotics Group and the Control Group (P > 0.05) (Table 2, Fig. 3).

### Effects of chronic stress on gut barrier function in mice

**Fig. 5:**
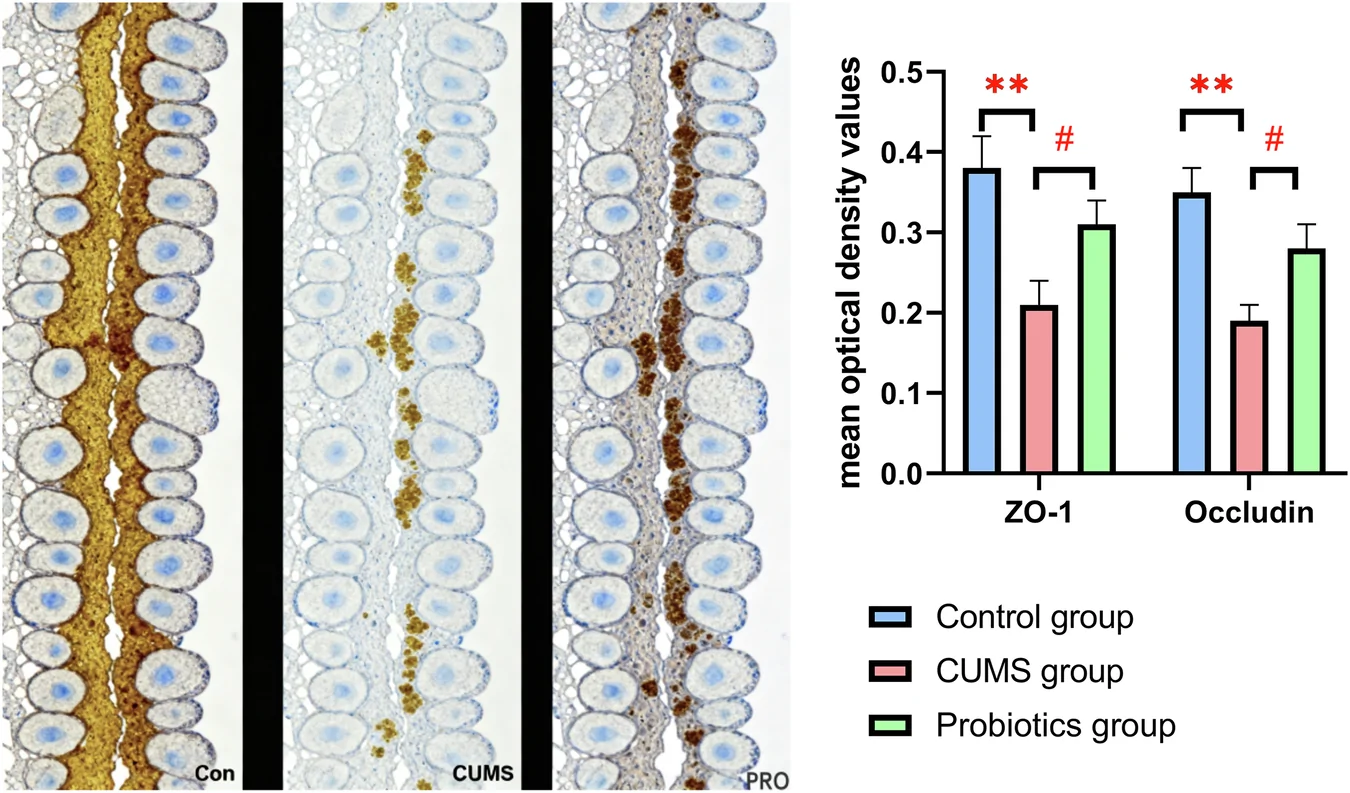
Effect of probiotics on the expression of tight junction proteins ZO-1 and Occludin in the colon of CUMS mice. Representative immunohistochemical staining images of ZO-1 and Occludin in colonic tissues. Brown-yellow staining indicates positive protein expression, primarily localized at the tight junctions between colonic mucosal epithelial cells; nuclei are counterstained blue. Compared with the Control group, the CUMS group exhibited noticeably reduced positive staining, the Probiotics group showed increased staining intensity compared to the CUMS group. Quantitative analysis of the mean optical density (MOD) is presented in Table 3.

Immunohistochemical results demonstrated that ZO-1 and occludin proteins were primarily expressed at the tight junction sites between colonic mucosal epithelial cells, exhibiting brown-yellow positive staining. Compared with the Control Group, the mean optical density values of ZO-1 and occludin proteins in the colonic tissue of the CUMS group were significantly decreased (P < 0.01). Both indicators in the Probiotics Group showed significant increases compared to the CUMS group (P < 0.05), although they remained lower than those in the Control Group (P < 0.05). The results indicate that CUMS stress can compromise gut barrier integrity in mice, while probiotic intervention partially restores gut barrier function (Table 3, Fig. 5).

**Table 3:** Comparison of mean optical density values in colonic tissue(x̄±s, n = 16).

	Control group	CUMS group	Probiotics group
ZO-1	0.38 ± 0.04	0.21 ± 0.03**	0.31 ± 0.03#
Occludin	0.35 ± 0.03	0.19 ± 0.02**	0.28 ± 0.03#

*P < 0.05 vs Control Group, **P < 0.01; #P < 0.05 vs CUMS group.

### Effects of chronic stress on neuroinflammatory cytokines in mice

#### Concentrations of inflammatory cytokines in hippocampus and prefrontal cortex

ELISA results showed that compared with Control Group, the concentrations of IL-1β, TNF-α and IL-6 in hippocampus and prefrontal cortex of CUMS group were significantly increased (P < 0.01); The concentrations of the aforementioned inflammatory cytokines in the Probiotics Group were significantly lower than those in the CUMS group (P < 0.05 or P < 0.01). Although the IL-6 concentration in the prefrontal cortex showed no statistically significant difference compared to the Control Group (P > 0.05), all other measured indices remained elevated relative to the Control Group (P < 0.05). Results indicate that CUMS stress induced neuroinflammatory responses, which were suppressed by probiotic intervention (Table 4).

**Table 4:** Comparison of inflammatory cytokine concentrations and inflammation-related proteins across three groups of mice (x ± s, n = 16, pg/mL).

	Control group	CUMS group	Probiotics group
Inflammatory Cytokine Concentrations in Hippocampal Tissue			
IL-1β	28.93 ± 3.21	65.78 ± 5.67**	42.34 ± 4.12##
TNF-α	32.45 ± 3.56	78.93 ± 6.78**	51.23 ± 4.89##
IL-6	25.67 ± 2.89	62.34 ± 5.32**	38.76 ± 4.12##
Inflammatory Cytokine Concentrations in the Prefrontal Cortex			
IL-1β	26.54 ± 2.98	58.93 ± 5.12**	39.87 ± 3.89##
TNF-α	30.12 ± 3.21	72.34 ± 6.23**	48.76 ± 4.56##
IL-6	23.45 ± 2.67	56.78 ± 4.89**	27.89 ± 3.01##
Inflammation-related Proteins in Hippocampal Tissues			
Iba-1/GAPDH	0.32 ± 0.04	0.87 ± 0.08**	0.41 ± 0.05##
iNOS/GAPDH	0.28 ± 0.03	0.76 ± 0.06**	0.35 ± 0.04#
COX-2/GAPDH	0.30 ± 0.03	0.82 ± 0.07**	0.38 ± 0.04#

** Compared with the Control Group, P < 0.01; #Compared with the CUMS group, P < 0.05, ##P < 0.01.

#### Expression of inflammation-related proteins in hippocampal tissue

**Fig. 6:**
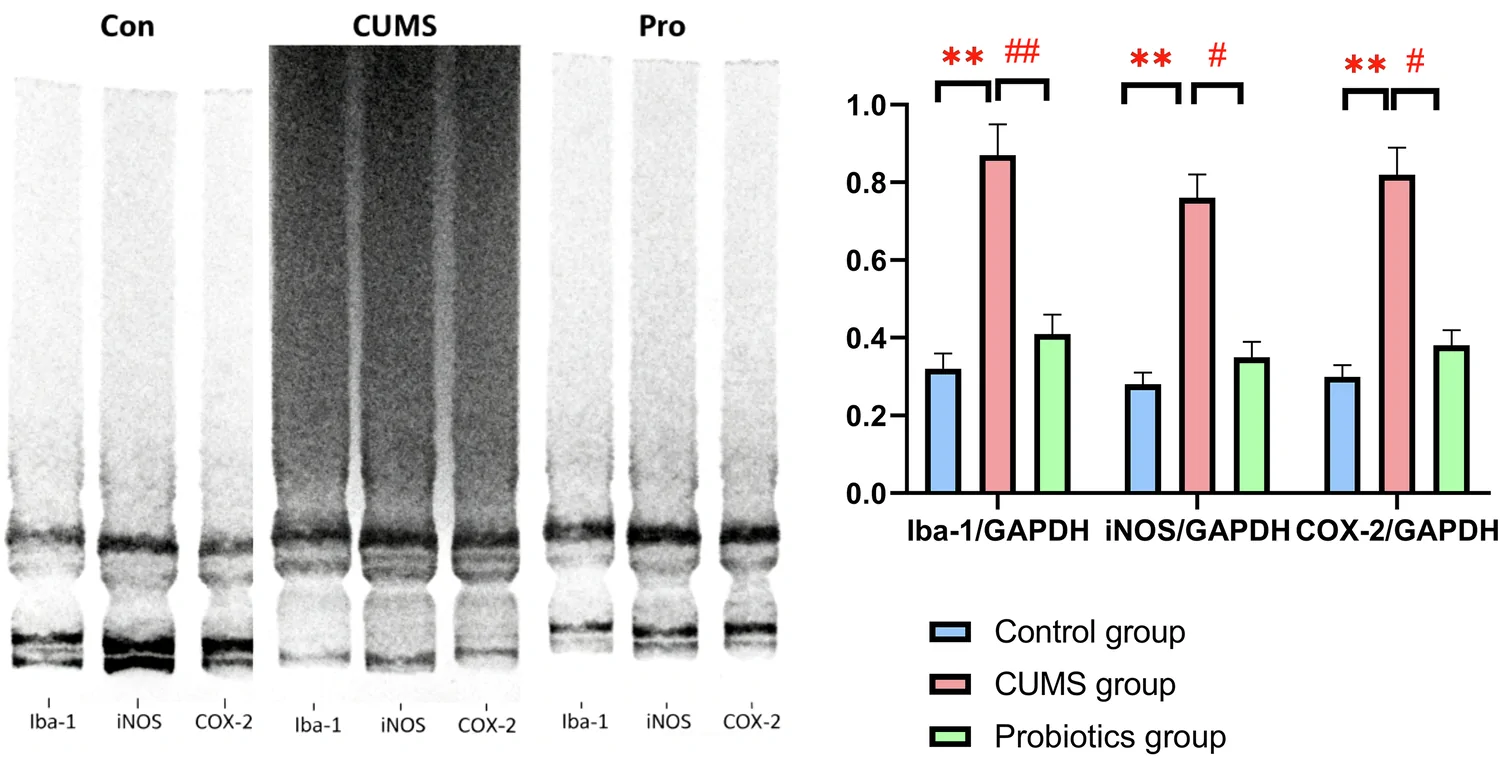
The expression of inflammation-related proteins in the hippocampus. Representative Western blot images analyzing Iba-1, iNOS, and COX-2 proteins in hippocampal lysates. GAPDH served as the loading control. Compared with the Control group, the CUMS group displayed notably increased band intensities for inflammatory markers (Iba-1, iNOS, and COX-2). The Probiotics group showed reduced band intensities compared to the CUMS group, appearing similar to the Control group. Detailed quantitative analysis of optical density is provided in Table 4.

Western blot results indicated that compared with the Control Group, the CUMS group exhibited significantly elevated relative expression levels of Iba-1 (a microglial activation marker), iNOS, and COX-2 proteins in hippocampal tissue (P < 0.01). The expression levels of these proteins in the Probiotics Group were significantly reduced compared with the CUMS group (P < 0.05 or P < 0.01), showing no statistically significant differences relative to the Control Group (P > 0.05). These results suggest that CUMS stress can activate microglia and inflammatory pathways, while probiotic intervention can inhibit microglial activation and suppress the activation of inflammatory pathways (Table 4, Fig. 6).

### Effects of chronic stress on morphology of hippocampal neurons in mice

**Fig. 7:**
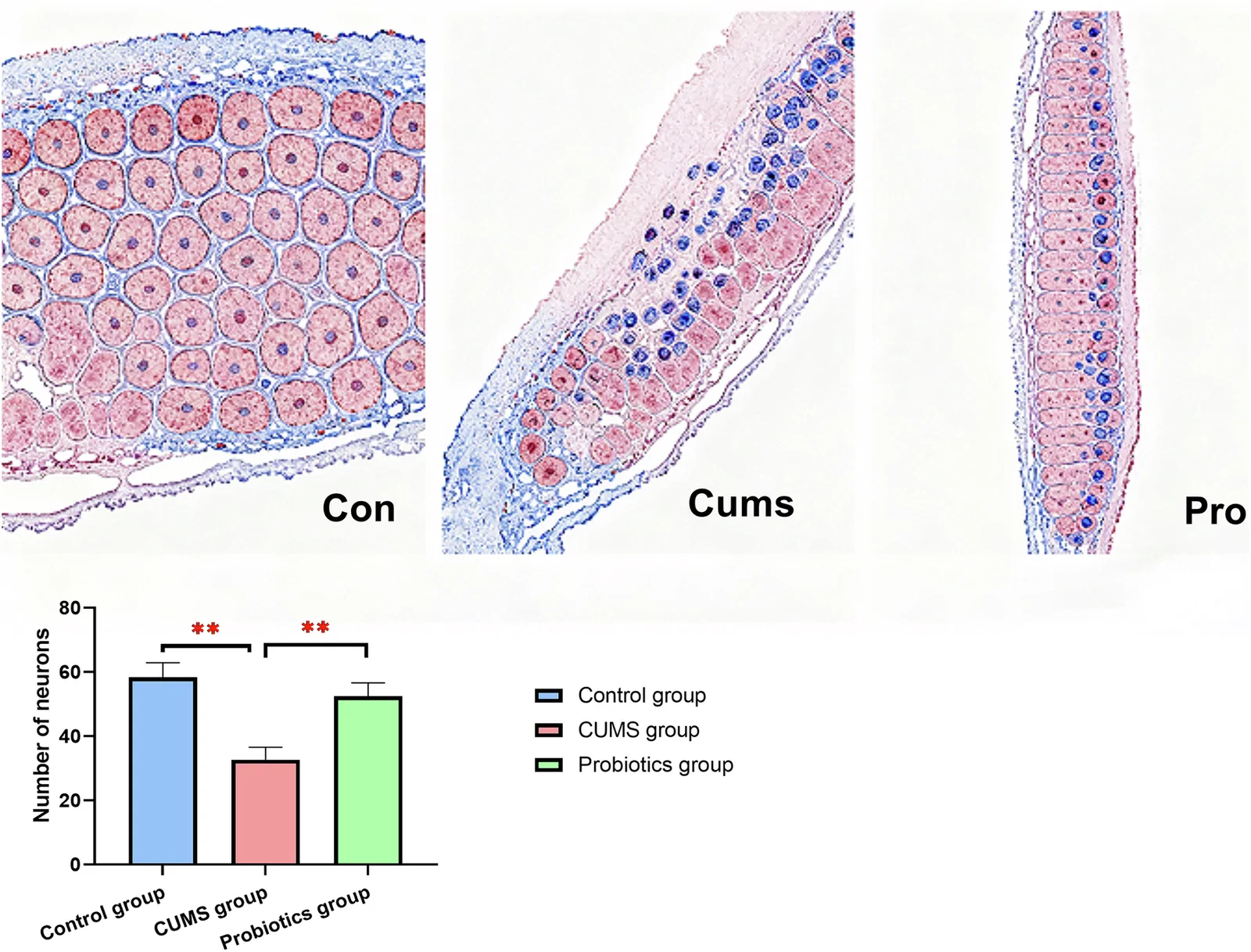
HE staining showing the effect of probiotics on neuronal morphology in the hippocampal CA1 region. Microscopic images of tissue sections from the hippocampal CA1 region. The Control group shows structurally intact neurons arranged in a dense and organized manner with clear nuclei. The CUMS group exhibits disorganized arrangement, sparse distribution, nuclear pyknosis, and deeply stained cytoplasm(**P < 0.01). In the Probiotics group, neuronal morphology is significantly improved compared to the CUMS group(##P < 0.01), with a more orderly arrangement and notably fewer pyknotic cells, resembling the Control group(På 0.05).

HE staining results demonstrated that in the Control Group, hippocampal CA1 region neurons exhibited well-organized and densely packed arrangements, with regular and centrally located nuclei and uniformly distributed cytoplasm, as the normal neurons number was 58.34 ± 4.56. The CUMS group displayed disorganized and sparsely distributed neurons, with some showing nuclear pyknosis and deeply stained cytoplasm, as the normal neurons number was 32.67 ± 3.89. A significant reduction in normal neuron count was observed (P < 0.01). The neurons in the Probiotics Group were arranged more orderly than those in the CUMS group with reduced nuclear pyknosis, and the number of normal neurons increased significantly (P < 0.01), showing no statistically significant difference from the Control Group (P > 0.05), as the normal neurons number was 52.45 ± 4.21. The results demonstrate that CUMS stress can induce damage to hippocampal neurons, while probiotic intervention protects hippocampal neurons from damage (Fig. 7).

## Discussion

### Characteristics and mechanisms of chronic stress-induced gut microbiota dysbiosis

The CUMS model was employed in this study to simulate chronic stress conditions. The results revealed a significant decrease in α-diversity of gut microbiota in the CUMS group, with statistically significant differences in β-diversity compared to the control group. These findings suggest that chronic stress can disrupt the stability and diversity of gut microbiota, which aligns with previous research [13]. Regarding microbiota composition, the CUMS group exhibited increased abundance of Firmicutes and decreased abundance of Bacteroidetes, with a significantly elevated F/B ratio. This trend has also been confirmed in patients with depression and anxiety disorders [14], potentially associated with Firmicutes’ higher efficiency in breaking down dietary fibers to produce energy, which may lead to metabolic disorders. At the genus level, the CUMS group showed reduced abundance of beneficial bacteria such as Bifidobacterium and Lactobacillus genus, while conditional pathogens like Escherichia genus demonstrated increased abundance. This further confirms that chronic stress can induce gut microbiota dysbiosis. Although there was a lack of Jaccard-based analysis, the multidimensional results still strongly support the study’s conclusions regarding β-diversity in gut microbiota abundance.

The mechanisms which chronic stress induces gut microbiota dysbiosis might be complex and interrelated. Chronic stress leads to HPA axis activation promoting cortisol secretion, which would directly affect gut microbiota growth by inhibiting beneficial bacteria like Bifidobacteria and Lactobacillus, while showing no significant suppression - or even promoting proliferation - of pathogenic bacteria such as Escherichia coli [15]; Stress-induced alterations in gastrointestinal motility (such as colonic hypermotility) could accelerate intestinal peristalsis, shortening gut microbiota colonization time and leading to altered microbial composition [16]; Stress would also reduce mucus secretion in the gut, diminishing the protective function of the mucus layer against microbiota. This leads to direct contact between microbiota and the intestinal mucosa, resulting in microbiota dysbiosis [17]. Probiotic Intervention in this study significantly restored gut microbiota diversity and composition, suggesting the reversible nature of microbiota dysbiosis under chronic stress conditions and providing experimental evidence for intervention strategies targeting gut microbiota.

### Association between gut microbiota dysbiosis and gut barrier impairment

The gut barrier, composed of intestinal mucosal epithelial cells and tight junctions (e.g., ZO-1, Occludin), serves as a crucial structure for maintaining intestinal homeostasis by preventing gut microbiota and their metabolites from entering the circulatory system [18]. This study found that the expression of ZO-1 and Occludin proteins in colonic tissue was significantly reduced in the CUMS group mice, indicating that chronic stress can compromise gut barrier integrity, consistent with previous research findings [19]. Probiotic intervention significantly increased the expression of ZO-1 and Occludin proteins, suggesting that probiotics can ameliorate chronic stress-induced disturbance in gut homeostasis by restoring gut barrier function.

A vicious cycle may form between gut microbiota dysbiosis and gut barrier impairment: On one hand, chronic stress-induced gut microbiota dysbiosis can reduce beneficial metabolites such as short-chain fatty acids. These fatty acids promote tight junction protein expression through AMPK signaling pathway activation, and their reduction directly contributes to gut barrier impairment [20]; On the other hand, gut barrier impairment can lead to microbial translocation, further exacerbating microbiota dysbiosis [21]. In this study, the CUMS group exhibited concurrent microbiota dysbiosis and intestinal barrier damage. Probiotic Intervention ameliorated both conditions, suggesting gut microbiota may influence gut barrier function by regulating tight junction protein expression. The specific molecular mechanisms require further investigation.

### Potential pathways of gut microbiota dysbiosis mediating neuroinflammation

Neuroinflammation is a pivotal mechanism of central nervous system (CNS) damage following chronic stress. Our results demonstrated significantly elevated concentrations of proinflammatory cytokines—including interleukin-1β (IL-1β), tumor necrosis factor-α (TNF-α), and interleukin-6 (IL-6)—in the hippocampus and prefrontal cortex of CUMS mice. Concurrent upregulation of the microglial activation marker Iba-1 and inflammatory pathway proteins (iNOS, COX-2) confirmed that chronic stress triggers a neuroinflammatory state. Probiotic intervention markedly reduced these inflammatory cytokine levels and protein expression, indicating that gut microbiota modulation can suppress neuroinflammation.

Existing evidence, together with our findings, points to microbial metabolite, immune, and neural pathways as potential routes through which gut dysbiosis mediates neuroinflammation. In the microbial metabolite pathway, dysbiosis may reduce the production of short-chain fatty acids (SCFAs). Butyrate, a key SCFA, acts not only as a primary energy source for colonocytes but also as a histone deacetylase (HDAC) inhibitor. Diminished butyrate may thus relieve epigenetic repression of inflammatory genes in microglia, aggravating neuroinflammation [22]. SCFAs normally cross the blood-brain barrier (BBB) to restrain microglial activation; their reduction could permit excessive microglial activity and the release of proinflammatory cytokines [20]. Simultaneously, an increased abundance of opportunistic pathogens can elevate pathogen-associated molecular patterns (PAMPs) such as lipopolysaccharide (LPS). LPS may translocate into circulation via a compromised intestinal barrier, provoking systemic low-grade inflammation that can cross the BBB and activate neuroinflammation [23]. Through the immune pathway, gut dysbiosis activates intestinal mucosal immunity, prompting cytokine release from gut immune cells that can reach the CNS via circulation, activate microglia, and induce neuroinflammation [24]. Via the neural pathway, dysbiosis can relay signals to the CNS through the vagus nerve, thereby activating microglia and promoting neuroinflammation [25]. In this study, probiotic intervention concurrently improved gut dysbiosis, intestinal barrier integrity, and neuroinflammation, supporting the involvement of these pathways in gut-mediated neuroinflammatory responses.

### Causality between neuroinflammation and anxiety- and depression-like behaviors

CNS dysfunction driven by neuroinflammation likely significantly contribute to anxiety- and depression-like behaviors. We observed disorganized and reduced neuronal numbers in the hippocampal CA1 region of CUMS mice. Given the hippocampus’s central role in emotional regulation, such neuronal damage can directly impair emotional control [26]. Beyond direct cellular injury, microglia-mediated synaptic pruning may represent a more refined pathological mechanism. Within the inflammatory microenvironment induced by chronic stress, activated microglia may excessively engulf normal excitatory synapses via the complement system, disrupting neural circuit connectivity and leading to behavioral abnormalities [27].

Stress response patterns also differ across brain regions. While this study focused on hippocampal neuronal damage, prior work indicates that under dysbiosis, the amygdala—a core region for anxiety and fear processing—often exhibits dendritic spine overgrowth and neuronal hyperexcitability. This structural imbalance, featuring hippocampal atrophy alongside amygdala hypertrophy, may be a key driver of anxiety-like behaviors [28]. Proinflammatory cytokines further exacerbate dysfunction by causing synaptic structural damage, reducing synaptic plasticity, and impairing neuronal signaling [29]. Moreover, these cytokines can inhibit tryptophan hydroxylase activity, lowering serotonin synthesis, while promoting indoleamine 2,3-dioxygenase activity to shift tryptophan metabolism toward kynurenine, thereby disrupting neurotransmitter balance [30]. In the present study, probiotic intervention significantly mitigated hippocampal neuronal damage, suggesting that probiotics may alleviate anxiety- and depression-like behaviors by inhibiting neuroinflammation, protecting hippocampal neurons, and maintaining homeostasis in the hippocampal-amygdala circuit.

### Conclusions

**Fig. 8:**
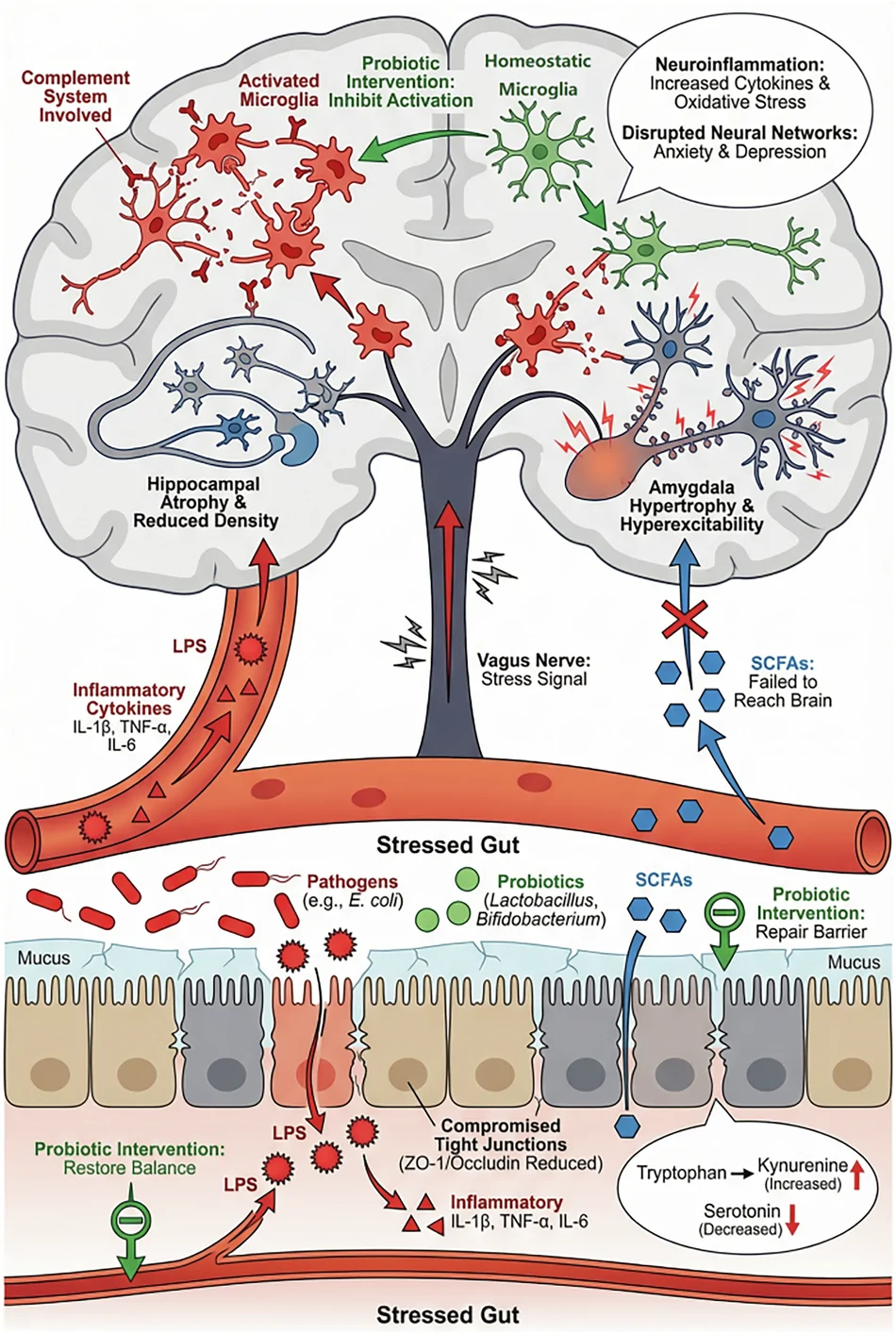
Schematic diagram illustrating the potential mechanism by which probiotics alleviate CUMS-induced depressive-like behaviors via the gut-brain axis. CUMS stress downregulates the expression of tight junction proteins (ZO-1 and Occludin) in the colon, compromising gut barrier integrity. This impairment may facilitate the translocation of inflammatory signals to the brain, triggering microglial activation (indicated by elevated Iba-1) and the release of inflammatory mediators (iNOS and COX-2) in the hippocampus, ultimately leading to neuronal damage. Probiotic intervention restores gut barrier function by upregulating tight junction proteins, thereby suppressing microglia-mediated neuroinflammation and exerting neuroprotective effects in the hippocampus.

Chronic stress can induce gut microbiota dysbiosis in mice (manifested as reduced diversity, elevated F/B ratio, decreased abundance of beneficial bacteria, and increased abundance of conditional pathogens) and gut barrier impairment (reduced expression of ZO-1 and Occludin proteins), subsequently activating neuroinflammatory responses (characterized by microglial activation and increased release of pro-inflammatory cytokines), significantly contribute to anxiety- and depression-like behaviors; Probiotic intervention can significantly ameliorate chronic stress-induced behavioral abnormalities by regulating gut microbiota composition, repairing gut barrier function, and inhibiting neuroinflammatory responses. These findings reveal the mechanistic role of the gut microbiota-gut-brain axis in stress-related psychiatric disorders, demonstrate the interaction between gut microbiota dysbiosis and neuroinflammation, and provide experimental evidence for developing novel gut microbiota-targeted intervention strategies[Fig. 8].

### Limitations

This study has certain limitations: 1. We did not measure concentration changes of gut microbiota metabolites (e.g., short-chain fatty acids, lipopolysaccharide), thus we could not determine the specific roles of metabolites in the interaction between microbiota dysbiosis and neuroinflammation; 2. We did not investigate specific molecular signaling pathways (e.g., TLR4/NF-κB pathway), making it difficult to elucidate the detailed mechanisms of neuroinflammatory activation; 3. The experiment exclusively utilized male mice and did not account for the potential impact of gender differences on experimental outcomes; 4. The lack of validation with clinical samples indicates that the clinical translatability of the experimental findings warrants further confirmation. 5. The lack of Jaccard-based analysis for the β-diversity assessment of gut microbiota. Future research should address these limitations by conducting in-depth investigations into the mechanistic role of the gut microbiota-gut-brain axis in stress-related psychiatric disorders.

## Data Availability

All the authors (xinhua.shen and xiaofeng.jiang) had full access to all data and takes responsibility for data integrity. Data generated during this study are available from the corresponding author on reasonable request.
